# Health-related quality of life in young adults with congenital central hypoventilation syndrome due to PHOX2B mutations: a cross-sectional study

**DOI:** 10.1186/s12931-015-0241-3

**Published:** 2015-06-30

**Authors:** Emilienne Verkaeren, Agnès Brion, Amélie Hurbault, Cécile Chenivesse, Capucine Morelot-Panzini, Jésus Gonzalez-Bermejo, Valérie Attali, Thomas Similowski, Christian Straus

**Affiliations:** AP-HP, Groupe Hospitalier Pitié-Salpêtrière Charles Foix, Département “R3S”, Service de Pneumologie et Réanimation Médicale, F-75013 Paris, France; AP-HP, Groupe Hospitalier Pitié-Salpêtrière Charles Foix, Département “R3S”, Service des Pathologies du Sommeil, F-75013 Paris, France; AP-HP, Groupe Hospitalier Pitié-Salpêtrière Charles Foix, Branche “Adultes” du Centre de Référence du Syndrome d’Ondine, F-75013 Paris, France; Sorbonne Universités, UPMC Univ Paris 06, INSERM, UMRS1158 Neurophysiologie respiratoire expérimentale et clinique, Paris, France; AP-HP, Groupe Hospitalier Pitié-Salpêtrière Charles Foix, Département “R3S”, Service d’Explorations Fonctionnelles de la Respiration, de l’Exercice et de la Dyspnée, F-75013 Paris, France

**Keywords:** Congenital central hypoventilation syndrome, Health-related quality of life, SF-36, Chronic respiratory insufficiency, Mechanical ventilation

## Abstract

**Background:**

Congenital central hypoventilation syndrome (CCHS) is a rare genetic disease due to PHOX2B mutations. CCHS patients suffer from many autonomic disorders, dominated clinically by defective ventilatory automatisms. From birth, the life of CCHS patients depends on ventilatory support during sleep, involving a high burden of care. Whether or not this impairs the quality of life of these patients during adulthood remains unknown.

**Methods:**

We applied the medical outcome study short form-36 (SF-36) to 12 CCHS patients aged 15–33 (9 women) at the time of their passage from pediatric to adult care. Scores for the SF-36 dimensions were compared to the age- and gender-matched French reference population after transformation into standardized Z-scores. The SF-36 physical component summary score (PCS) and mental component summary score (MCS) were compared to American reference values.

**Results:**

Median Z-scores were significantly different from zero for PF (physical functioning, *p* = 0.020) and GH (general health perception, *p* = 0.0342) and for PCS (*p* = 0.020). The other physical dimensions (RP, role limitation due to physical function; BP, bodily pain) and the mental dimensions (VT, vitality; SF, social functioning; RE, role limitation due to emotional function; MH, mental health) and MCS were not altered.

**Conclusions:**

We conclude that, despite the physical constraints imposed by CCHS and its anxiogenic nature, this disease is associated with an impairment of health-related quality of life in young adults that remains moderate. Whatever the underlying explanations, these results convey hope to parents with a child diagnosed with CCHS and for patients themselves.

## Introduction

Congenital central hypoventilation syndrome (CCHS) is a very rare genetic disease (1/200 000 births in France [1] related to mutations of the PHOX2B gene [2–5]. It involves multiple autonomic nervous system disorders and is predominantly marked by impaired ventilatory function due to defective chemosensitivity: profound sleep-related hypoventilation makes patients dependent on ventilatory support whenever they go to sleep. Hypoventilation can sometimes extend to wakefulness. Ventilatory support is therefore the cornerstone of CCHS management. During the first years of life, ventilatory support is administered by tracheotomy, with the associated constraints [6]). Mask ventilation can be instituted around age 6 to 8, but is also associated with specific constraints [7]. Ventilatory support notably affects the patient’s and family’s lifestyle [8]. In addition, CCHS patients may present cardiac conduction disorders [7, 9, 10], severe constipation with Hirschsprung’s disease [7], gastroesophageal reflux, thermal dysregulation, orthostatic hypotension [11], ophthalmologic disorders [7, 12], or neural crest tumors [7]. These abnormalities generate symptoms and may require specific treatments. Regular hospital assessments are recommended [7].

All these medical constraints can have a negative impact on quality of life, as in the case of any chronic disease. In addition, CCHS limits physical activities because of the risk of fatal “silent” hypoxic accidents, and is anxiogenic for patients and their families [8]. CCHS therfore satisfies the World Health Organization definition of a disability [13].

The transition of CCHS patients from childhood to adulthood has not been extensively described. Their disability could possibly interfere with their quality of life. The objective of this study was to assess this particular aspect of the health of young adults with CCHS.

## Material and methods

### Patients

The study pertains to the first 12 patients with CCHS due to PHOX2B mutations who were referred to the adult division of the French Reference Center for CCHS (9 women, 3 men; median age 24 —15 to 33—; 3 patients less than 18). It was approved by the Institutional Review Board of the French learned society for respiratory medicine -*Société de Pneumologie de Langue Française*-. Patients gave consent to participate.

CCHS had been diagnosed at birth in 8 cases and before 1 year of age in four. PHOX2B abnormalities consisted of alanine expansion in 11 cases (25 alanines, *n* = 3; 26, *n* = 3; 27, *n* = 2; 29, 30 and 31 in each of the remaining cases) and a frameshift mutation in one case. All patients had been tracheotomized before age one, seven had been transitioned to noninvasive ventilation (at median age of 16.5 years −8 to 22-), five were still tracheotomized at the time of the study. Only one patient was permanently dependent on ventilatory support. No patient required diaphragm pacing at the time of the study (but 2 were implanted subsequently).

Three patients had documented mild-to-moderate pulmonary artery hypertension, five had gastroesophageal reflux requiring medical treatment, two had Hirschsprung’s disease (having required extensive colectomy in one case). Two patients had undergone strabismus corrective surgery, five suffered from convergence deficiency, and six had refraction abnormalities (some of these patients have been described in [12]).

All patients had attended school since early childhood. At the time of the study, six were still attending secondary school, two had reached the level of the French General Certificate of Education (“baccalauréat”), and 1 was a postgraduate university student. Two patients had a full-time job and one was unemployed.

Six patients still lived with their parents, five lived away from home (including one in a couple), and one was institutionalized. All patients were childless at the time of the study (but two had children after the study; one woman had two children, both free of CCHS —the couple refused prenatal diagnosis—; one man had one child with a positive prenatal diagnosis).

### Questionnaires

The questionnaires were administered during the patient’s very first visit to the adult division of the reference center.

#### Quality of life

Health-related quality of life was assessed with the validated French version of the medical outcome study short form-36 (SF-36) self-administered questionnaire. SF-36 examines 8 dimensions (PF, physical functioning; RP, role limitation due to physical function; BP, bodily pain; GH, general health perception; VT, vitality; SF, social functioning; RE, role limitation due to emotional function; MH, mental health). Each of the eight dimensions is scored from 0 (worst quality of life) to 100 (best). Two summary scores are also calculated, the Physical Component Summary (PCS) and the Mental Component Summary (MCS). French values obtained in the general population over the age of 14 were used as reference values for the 8 SF-36 dimensions [14]. American reference values were used for PCS and MCS [15], in the absence of other available data.

#### Anxiety and depression

The patients also filled in the Beck Depression Inventory (BDI, version I in one patient, version II in all other patients) [16]) and the State Trait Anxiety Inventory (STAI) [17].

### Data management and statistical analysis

For each SF-36 dimension and PCS-MCS, observed values were transformed into the corresponding Z-scores (observed value minus arithmetic mean [in the age- and gender-matched general population] divided by the standard deviation). We considered that a Z-score above −1.64 (5th percentile of the reference population) did not correspond to a clinically significant alteration of the dimension considered, a Z-score between −1.64 and −1.96 (5th to 2.5th percentile) corresponded to moderate alteration, a Z-score between −1.96 and −2.33 (2.5th to 1st percentile) corresponded to severe alteration, and a Z-score below −2.33 (1st percentile) corresponded to very severe alteration. This type of stratification has been used before for SF-36 data [18], the choices of 5th, 2.5th and 1th percentiles being widely accepted.

Statistical analysis was performed with Prism® version 5 (GraphPad Software, Inc., La Jolla, CA, USA) and an online R module for Cronbach’s alpha coefficient [19]. Because the Z-score distributions did not consistently pass normality testing, the data are summarized as medians and interquartile ranges. The frequencies of a Z-score below −1.64, below −1.96 and below −2.33 were compared among SF-36 dimensions by applying a Chi-2 test to a 2×8 contingency table, and between PCS and MCS by applying a Chi-2 test to a 2×2 contingency table. The median of the Z-score distribution was compared to a theoretical zero median using Wilcoxon’s signed rank test. Cronbach’s α coefficient was used to evaluate the internal consistency of the SF-36 Z-score transformation [20]. P less than 5 % was considered significant.

## Results

### SF-36

All patients fully completed the SF-36 questionnaire (no missing data).

#### SF-36 dimensions

**Fig. 1:**
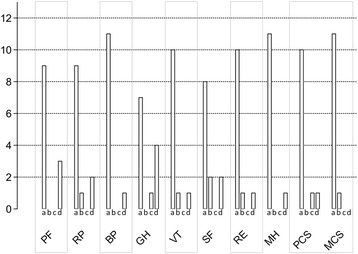
Number of patients in each Z-score category for the SF-36 components and component summaries. a: Z-score above −1.64, no significant alteration of quality of life; b: Z-score from −1.64 to −195, moderate alteration of quality of life; c: Z-score from −1.96 to −2.32, severe alteration of quality of life; d: Z-score 2.33 and below, very severe alteration of quality of life. PF, physical functioning; RP, role limitation due to physical function; BP, bodily pain; GH, general health perception; VT, vitality; SF, social functioning; RE, role limitation due to emotional function; MH, mental health; PCS, physical component summary; MCS, mental component summary

Z-scores are summarized in Fig. 1. The frequencies of a Z-score below −1.64, below −1.96, or below −2.33, were not significantly different across dimensions (*p* = 0.764, *p* = 0.586, *p* = 0.816, respectively). The median Z-score was significantly different from zero for PF (physical functioning, *p* = 0.020) and GH (general health perception, *p* = 0.034) (Table 1). Cronbach’s alpha was 0.89, indicating satisfactory internal consistency [21].

**Table 1:** Absolute values and Z-scores for the 8 SF-36 dimensions

SF-36 dimensions	Rating	Z-score	Comparison of Z-score with theoretical zero median *(reference population, Wilcoxon’s signed rank test)*
	Median [interquartile range]	Median [interquartile range]	
PF (physical functioning)	87.5 [68.8/95]	−0.541 [−2.283/−0.283]	*p* = 0.020
RP (role limitation due to physical function)	100 [50/100]	0.3445 [−1.688/0.489]	*p* = 0.724
BP (bodily pain)	84 [65/97.5]	0.252 [−0.674/0.700]	*p* = 0.844
GH (general health perception)	54.5 [30.5/82]	−1.324 [−2.531/−0.501]	*p* = 0.034
VT (vitality)	50 [37.5/73.8]	−0.712 [−1.560/0.7120]	*p* = 0.203
SF (social functioning)	81.25 [37.5/100]	0.009 [−1.886/0.892]	*p* = 0.610
RE (role limitation due to emotional function)	100 [75/100]	0.492 [−0.434/0.620]	*p* = 0.665
MH (mental health)	70 [68/80]	0.293 [−0.302/0.817]	*p* = 0.519

*PF* physical functioning, *RP* role limitation due to physical function, *BP* bodily pain, *GH* general health perception, *VT* vitality, *SF* social functioning, *RE* role limitation due to emotional function, *MH* mental health

#### SF-36 summary components

Only one patient (#8) had a Z-score below −1.64 for PCS and for MCS. The median Z-score was significantly different from zero for PCS (*p* = 0.020), but not for MCS (Table 2).

**Table 2:** Absolute values and Z-scores for the 2 SF-36 summary scores

SF-36 summary scores	Rating	Z-score	Comparison of Z-score with theoretical zero median *(reference population, Wilcoxon’s signed rank test)*
	Median [interquartile range]	Median [interquartile range]	
PCS (physical component summary)	49.8 [42.9–55.3]	−0.377 [−1.194–0.243]	*p* = 0.020
MCS (mental component summary)	48.4 [39.9–53.49]	−0.01 [−0.790–0.629]	*p* = 0.850

*PCS* physical component summary; reference value in gender-/age-matched population 53.2 [53.0–54.0]

*MCS* mental component summary; reference value in gender-/age-matched population 47.1 [44.3–49.4]

### BDI

**Fig. 2:**
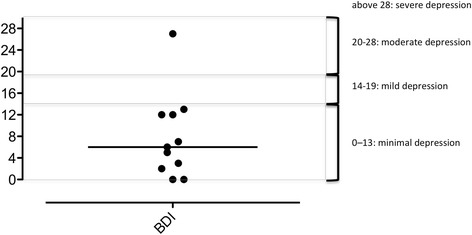
Beck depression inventory scores (BDI-II) in 11 of the 12 patients of the cohort (one patient was evaluated with the first version of the BDI inventory)

One patient (#8) had a BDI-II score of 27, at the upper limit of “moderate” depression and close to “severe” depression, and one had a BDI score associated with moderately severe depressive symptoms (patient #1, BDI-I = 9). The other patients were classified in the “no depression or minimal depression” range (BDI-II from 0 to 13) (Fig. 2).

### STAI

**Fig. 3:**
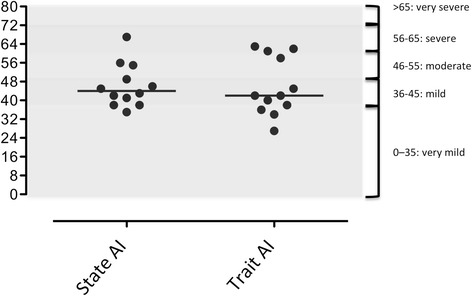
State trait anxiety inventory scores (state anxiety on the left, trait anxiety on the right) in the 12 patients of the cohort

According to French references [22, 23], one patient had very severe anxiety (patient #2), 1 patient had severe anxiety (patient # 1), 3 patients had moderate anxiety (#4, 8, 11), and 6 patients had mild or very mild anxiety (Fig. 2). Trait anxiety was very mild in two patients (#3 and 12), mild in 6 (#5, 6, 7, 9, 10 and 11), and severe in 4 (#1, 2, 4, 8) (Fig. 3).

## Discussion

The 12 CCHS patients who participated in this study (nine young adults, three late adolescents) described alterations in health-related quality of life. These alterations were however relatively moderate (five patients had Z-scores within the normal range for all SF36 dimensions; 3 patients had Z-scores outside this range for only 1 dimension; only two SF-36 dimensions -“physical functioning” and “general health”- and the physical component summary score significantly differed from reference values) (Table 1). Depression was rarely reported (Fig. 2), but anxiety was frequent (Fig. 3).

### Study weaknessses and strengths

The main weakness of the study resides in the small size of the population, but it should be kept in mind that less than 70 PHOX2B-CCHS patients were known in France and that we included all the patients who could reasonably qualify as “young adults” at the relevant time. Because of this size limitation, it must be kept in mind that the lack of significant differences that we found regarding most of the SF36 Z-scores and zero could proceed from lack of statistical power. Also because of the small size of the study, it was not possible to conduct any explanatory analysis to identify the drivers of the alterations in quality of life. These drivers can be extremely diverse (possibly including the specific PHOX2B genotype and the related phenotype -respiratory and otherwise-, parental marriage status, proband’s marriage status, socio-economic status of the nuclear family, parental viewpoint on life in general, age at mask introduction, rigor of artificial ventilation management prescription, compliance with recommended artificial ventilation, etc.), and we acknowledge that our study was not designed or powered to tackle this issue. Rather, its aim was, at the moment where the patients were referred to our center, to get a general picture of their quality of life. Further studies and comparisons with other countries will therefore be needed. However, our study also has strengths. It pertains to a well-identified population of patients. It uses very generic tools that are simple, widely applicable and widely validated. It provides an information that is by essence novel.

### Comparison with other conditions

CCHS is a form of chronic respiratory insufficiency that is most particular in that it is not associated with dyspnea. This probably accounts for the absence of abnormalities of the SF-36 “bodily pain” dimension in our patients, which contrasts with studies pertaining to forms of chronic respiratory insufficiency [24–32]. In these studies, SF-36 physical functioning and physical summary score were severely altered [26, 30–32] and abnormalities in “mental” dimensions were also observed [27, 32]. This is another major difference with our results. Of note, the above studies concerned patients older than ours, which can be a confounder. SF-36 abnormalities are much more severe in young adults with cystic fibrosis than in our patients [33], again probably because of intense “respiratory suffering”. In contrast, Duchenne patients who have become completely dependent on mechanical ventilation express positive views of their situation [34] possibly because, at this stage, they are no longer exposed to exercise-related dyspnea. Many chronic non-respiratory diseases (sickle cell disease, chronic renal insufficiency, type I diabetes, HIV infection, narcolepsy and idiopathic hypersomnia all impair health-related quality of life [35–39]) or sensory defects (deafness or blindness) [40] affecting young adults also impair their quality of life. SF-36 scores are generally lower in these clinical settings than in our patients.

### Interpretation

As mentioned before, the small size of the study population does not allow sophisticated statistical approaches. Patient #8 (severe hypoventilation; tracheotomy; moderate pulmonary hypertension) exhibited the most diffuse and most severe SF-36 abnormalities. This patient also had a high BDI score compatible with clinical depression that could have an intrinsic impact on quality of life. In contrast, patient #7 reported 4 abnormal SF-36 dimensions with low BDI and STAI scores. This patient had recently suffered a sports injury, the orthopedic consequences of which may have impacted quality of life independently of CCHS. Of note, the patient with the most severe hypoventilation, requiring permanent mechanical ventilation (#12), reported only two abnormal SF-36 dimensions. The lack of relationship between disease severity and SF-36 abnormalities has been previously described [33, 37].

Even though some individuals presented some severely abnormal scores, the cohort as a whole exhibited relatively moderate SF-36 abnormalities in the physical domain. Of importance, no abnormalities were observed in the mental domain despite a high frequency of sometimes severe anxiety (Fig. 3). Our patients all had very early childhood diagnosis and had lived with their ventilators from birth. They therefore had had times to develop a personal set of values and to conceptualize their well-being and mental health with respect to their condition, without reference to a previous better condition. Preserved quality of life has been described in adult patients with severe congenital heart disease [41], who also have no experience of a disease-free period of life. Our patients were all fully independent regarding the management of their ventilatory support, and had been trained to be independent since childhood. This might have given them a feeling of control, minimizing the psychological impact of ventilator dependency. In support of this view, several patients reported that they wanted to be “like other people” and “push themselves to the limits”. All but one of the patients were adequately inserted in school or at work, another factor that limits the disability related to a chronic disease (consistent with data observed in children in [8]). Two of our patients decided to have children. Finally, CCHS patients in France are managed by a limited number of specialists, according to strict and standardized procedures. They are also closely integrated in a community of parents and patients with the activities of “*Association Française du Syndrome d’Ondine*”, all of which may be a source of reassurance and a feeling of being “normal”.

## Conclusions

A diagnosis of CCHS at birth is devastating for the child’s parents. Although not specifically assessed up until now, this diagnosis obviously imposes a considerable burden on the families [8], not only in terms of physical care and daily stress, but also in terms of their concerns about the future well-being of their child. By showing that alterations in quality of life are moderate in young adults having suffered from CCHS since birth, our study brings a positive message to the parents of CCHS patients and to the patients themselves, keeping in mind that the prospect of a satisfactory quality of life does not change the fact that CCHS is a potentially lethal disorder and that our results do not bring any information regarding vital prognosis at the time of diagnosis. It would be interesting to perform similar studies in CCHS patients from other countries, and, in our setting, to follow the course of quality of life in adult CCHS patients with aging and the corresponding changes in social life.

## Abbreviations

BP: Bodily pain (SF-36 component)
CCHS: Congenital central hypoventilation syndrome
DBI: Beck Depression Inventory
GH: General health perception (SF-36 component)
MCS: Mental component summary score (SF-36 summary score)
MH: Mental health (SF-36 component)
PCS: Physical component summary score (SF-36 summary score)
PF: Physical functioning (SF-36 component)
RE: Role limitation due to emotional function (SF-36 component)
RP: Role limitation due to physical function (SF-36 component)
SF-36: Medical outcome study short form-36
SF: Social functioning (SF-36 component)
STAI: State Trait Anxiety Inventory
VT: Vitality (SF-36 component)
